# Polymorphism IL-1RN rs419598 reduces the susceptibility to generalized periodontitis in a population of European descent

**DOI:** 10.1371/journal.pone.0186366

**Published:** 2017-10-12

**Authors:** Francisco Mesa, Esperanza Lanza, Llenalia García, Rafael Marfil-Alvarez, Antonio Magan-Fernandez

**Affiliations:** 1 Periodontology Department, School of Dentistry, University of Granada, Granada, Spain; 2 Private Periodontology and Implant Practice, Malaga, Spain; 3 SEPLIN Statistical Solutions, Spin-Off University of Granada, Granada, Spain; The Ohio State University, UNITED STATES

## Abstract

Interleukin (IL) 1-ra is a potent endogenous competitive inhibitor of IL-α and β and has an anti-inflammatory role. Study objectives were: 1) to assess the associations of IL-1RN genetic single nucleotide polymorphism (SNP) (rs419598) with generalized chronic periodontitis (GCP), generalized aggressive periodontitis (GAgP), and absence of periodontitis and 2) to assess its association with the load of five periodontopathogenic bacteria and periodontal clinical variables. A cross-sectional analytic study was conducted in 123 patients with GCP, 60 patients with GAgP, and 20 controls. Reverse hybridization PCR was used for genotyping analysis to detect SNPs in IL-1A (rs1800587), IL-1B (rs1143634), and IL-1RN (rs419598) genes and for determination of the load of five periodontopathogenic bacteria. The severity and extension of periodontitis were assessed. Multinomial logistic regression and mediated regression analyses were performed. Considering results for GCP and GAgP patients together, the presence of polymorphism in IL-1A and/or IL-1B gene was associated with a higher likelihood of periodontitis, (OR = 8.11; 95%CI [1.85–35.48]), but this likelihood was reduced when IL-1RN polymorphism was also present, (OR = 5.91; 95%CI [1.08–32.27]). IL-1RN polymorphism was significantly associated with lower counts of red complex bacteria, specifically *Porphyromona gingivalis*, *Tannerella forsythia*, and *Prevotella intermedia*, which were associated with improved clinical outcomes. The polymorphic expression of IL-1RN (rs419598) gene may be associated with a reduced susceptibility to GAgP and GCP in populations of European descent. This effect may be mediated by a decreased load of *Porphyromona gingivalis*, *Tannerella forsythia*, and *Prevotella intermedia*.

## Introduction

The Interleukin 1 (IL-1) family comprises three molecules, two agonists (IL-1α and IL-1β) and a specific IL-1 receptor antagonist (IL-1ra), as well as two membrane receptors (IL-1RI and IL-1RII). These cytokines have a crucial role in the innate immune system and regulate functions of the adaptive immune system [1], decisively mediating in the inflammatory response to noxious agents and in tissue damage [2].

Over the past 34 years, IL-1α and IL-1β have been reported to play an important pro-inflammatory role in multiple human diseases, including infectious diseases. Their development is favored by the overproduction of IL-1α, β and/or underproduction of IL-1ra, and the therapeutic administration of IL-1ra, as recombinant protein, has proven efficacious to prevent tissue damage[1]. IL-ra is a potent endogenous competitive inhibitor of IL-α and β and exerts anti-inflammatory effects [3]. After injection of LPS in healthy volunteers, a peak of plasma IL-1β was observed at 2 hours, with a concentration of approximately 80 pg/ml, followed at 3–6 hours by a peak of IL-1ra at almost 100-fold higher concentrations[4]. These elevated IL-ra concentrations are primarily produced by the liver as an acute-phase protein [5], influencing the IL-1β /IL-1ra ratio at local level. IL-1ra concentrations must be at least 100-fold higher than IL-1β concentrations to functionally inhibit the biologic effects of IL-1β on target cells; hence, an abundant production of IL-1ra is necessary in local tissues to block the effects of IL-1β [6].

Various studies found that allelic variations in the genes that encode these cytokines can affect susceptibility to periodontitis and its progression [7–9]. Two recent meta-analyses reported distinct effects on susceptibility to chronic periodontitis of single nucleotide polymorphism (SNP) rs1143634 *versus* rs16944 in IL-1B gene [10, 11]. Two meta-analyses on the same SNP in IL-1A gene, rs1800587, found an association with chronic [12] but not aggressive [13] periodontitis.

The polymorphism of the IL-1RN gene most widely studied in association with periodontitis is the variable number tandem repeat (VNTR), a penta-allelic 86-bp polymorphism (rs2234663). However, the only published meta-analysis described contradictory results according to the ethnicity of the study population and the type of periodontitis, and no conclusion could be established [14]. Contradictory findings were also reported by the only three publications on the relationship between periodontitis susceptibility and SNP (thymine/cytosine) at position +2018 of the RN gene (rs419598)[9, 15, 16]. Hence, there is inconsistent evidence on IL-1 genetic risk factors for periodontitis.

Recently published data on the red complex load and clinical variables in the same patient sample demonstrated an association between higher red complex count and worse periodontal outcomes in both periodontitis types [17]. Our study hypothesis was that the expression of SNPs in the RN gene may be associated with reduced clinical susceptibility to generalized chronic (GCP) and/or aggressive periodontitis (GAgP). The objectives of this study were: 1) to compare the frequency of several SNPs in the IL-1 family (IL-1A (rs1800587), IL-1B (rs1143634), and IL‐1RN (rs419598)) among patients with GCP, patients with GAgP, and healthy controls; and 2) to assess the association of this polymorphism with clinical periodontal variables and the load of five periodontopathogens, including red complex bacteria.

## Materials and methods

### Study design and population

STROBE (Strengthening the Reporting of Observational Studies in Epidemiology) guidelines were followed [18] in this cross-sectional analytic study of consecutive patients under treatment for periodontitis at a private clinic (Malaga, Spain). Diagnostic criteria for both GAgP and GCP were: probing pocket depth (PPD) ≥4 mm and clinical attachment (CA) loss ≥3mm at the same site in more than 30% of residual teeth [19]; bleeding on probing in affected sites, and bone loss confirmed by periapical radiological examination. Differential diagnostic criteria were: non-contributory medical history, self-reported familial aggregation [20], and age ≤35 years [21] for GAgP; and were age >35 years with no self-reported familiar aggregation for GCP. A control group with a similar sex distribution compared to both periodontitis groups was formed of non-smokers attending the clinic for implant treatment and found to be periodontally healthy by clinical examination. Controls were only used for the comparison of genetic polymorphism expression. Study exclusion criteria for all groups (GAgP, GCP, and controls) were: antibiotic or anti-inflammatory treatment in the previous 6 months or periodontal treatment in the previous 12 months, <14 teeth in the mouth, any systemic disease, pregnancy, breastfeeding, the presence of implant-retained prostheses and/or partial or unitary fixed prostheses, and non-European descent.

A minimum of 47 patients with GAgP and 103 with GCP was estimated to be necessary to compare clinical variables associated with IL-1 genotype polymorphisms between GCP and GAgP patients with statistical power of 0.8 and standardized mean difference of 0.5 between the groups for the main quantitative outcomes. Twenty controls were estimated to be sufficient to test differences in the proportions of cases with genotype polymorphism of 30%, with a statistical power of ≥0.8 and a significance level of 5% (using logistic models, assuming binomial distribution with a probability of 0.5, the minimum total sample size of cases and controls was 81).

Informed written consent was obtained from all participants in the study, which complied with the principles of the 2013 Helsinki Declaration and was approved by the research ethics committee of the School of Dentistry of the University of Granada (FOD/UGR/65/2015, 30/11/2015).

### Socio-demographic and clinical variables

Data were gathered on sex, age, smoking habit (n° cigarettes/day), and family history of periodontitis. Periodontal examinations were conducted by a single examiner (E.L.), who used a periodontal probe (PCP-UNC15, Hu-Friedy, Chicago, IL, USA) to probe six sites in each tooth, determining the PPD and CA loss (in mm), the gingival bleeding index (GBI) [22], and the plaque index [23]. Intraclass correlation coefficients for PPD measurements were 0.80 for intra-examiner agreement and 0.78 for inter-examiner agreement (comparing with examinations by F.M.), a high degree of agreement. The severity of periodontitis was evaluated using a modification of the Periodontal Inflammatory Severity Index [24], referred to hereafter as the PISIM. The PISIM score is the sum of the product of the number of sites and the PPD at each site divided by the number of remaining teeth (PISIM = ∑ (d_i_ n_i_)/t), where “i” is the site, “d” is the PPD of the site in mm, “n” is the absolute frequency of the sites, and “t” is the number of remaining teeth. The degree of periodontitis was expressed as the number of sites with CA loss ≥3 mm.

### Microbiological analysis

Subgingival plaque samples were taken from the deepest pocket in each quadrant that also showed gingival redness and swelling, using two ISO n° 40 blotting paper tips [25]. Samples were placed in a 1.5-ml test tube (Test tube, Eppendorf AG, Hamburg, Germany) and kept at 4°C until DNA extraction within 24 h by the Echevarne Microbiology Laboratory (Malaga, Spain), using a preparation kit (High Pure PCR Template Preparation Kit, Roche, Mannheim, Germany) and following the manufacturer's recommendations. A PCR test (MicroIDent, Hain Lifescience, Nehren, Germany) was performed, using primers reported by Ashimoto et al. [26] for *Porphyromona gingivalis (P*. *gingivalis)*, *Tannerella forsythia (T*. *forsythia)*, *Treponema denticola (T*. *denticola)*, and *Prevotella intermedia (P*. *intermedia)* and those reported by Tran and Rudney [27] for *Aggregatibacter actinomycetemcomitans* (*A*. *actinomycetemcomitans)*. Reverse hybridization was then carried out according to the manufacturer’s instructions, strips were then scanned using image processing software (Adobe Photoshop Elements, Adobe Systems, San Jose, CA, USA) followed by measurement of the luminescence of bands. The range of the white background of the strips and the conjugate control was set at 100%, and the value of each band was expressed as the percentage of control staining. The semi-quantitative methodology was adjusted (sensitivity) by using the following species at concentrations of 10−10^8^ bacteria: *A*. *actinomycetemcomitans* ATCC 33384, *P*. *gingivalis* ATCC 33277, *T*. *forsythia* ATCC 43037, *T*. *denticola* ATCC 35405, *P*. *intermedia* ATCC 25611. Sensitivities for microIDent® results were between 10^3^ and10^4^ bacteria. No cross-reactivity among these species was found. The specificity of microIDent® was previously established by database analysis [26–28]. The microbiological analysis protocol was described in detail in a previous publication [17].

### Genetic analysis

Samples were gathered by swabbing the buccal mucosa and dorsum of the tongue with sterile cotton swabs, and DNA extraction was carried out with the same technique as for plaque samples. Multiplex PCR of 16s rDNA and simultaneous reverse hybridization using semiquantitative DNA–Strip® technology were performed (GenoType IL-1, Hain Lifescience, Nehren, Germany), studying IL-1A -C889T (rs1800587), IL-1B +C3953T (rs1143634), and IL‐1RN +T2018C (rs419598). PCR amplification was performed out in 50 μL of reaction volume comprising 5 μL of template DNA and 45 μL of reaction mixture containing 35 μL PNM, 5 μL 10× PCR buffer, 2.5 μL 25 mmol/L MgCl2, and 1 U taq polymerase (Fermentas, Thermo Fisher Scientific, Waltham, MA, USA). PCR cycling was done in a thermal cycler (Mastercycler, Eppendorf AG, Hamburg, Germany) as follows: initial denaturation step at 95°C for 5 min; 10 cycles at 95°C for 30 s and at 60°C for 2 min; 20 cycles at 95°C for 10 s, at 55°C for 30 s, and at 72°C for 30 s; and a final extension step at 72°C for 10 min according to recommendations of the taq polymerase manufacturer. Reverse hybridization was then performed according to the manufacturer’s instructions. SNPs were considered heterozygous when both alleles were detected or homozygous when the polymorphic allele alone was present.

### Statistical analysis

Descriptive statistical analysis was performed to summarize the data by study group, calculating the frequencies and percentages for categorical variables and using chi-square tests to check their independence. Logistic regression models were applied to evaluate the association of genotype polymorphisms with periodontitis, obtaining the adjusted OR by age and sex with 95% confidence intervals. Multinomial logistic regression models were also constructed to study the association of genotype polymorphisms with specific periodontitis types (aggressive or chronic periodontitis vs. controls) [29]. Multivariate linear regression analysis was performed to analyze the association of genotype polymorphisms with clinical variables and bacterial loads in the periodontitis patients, examining the effect of bacterial loads as a mediated variable between genotype polymorphism and clinical response [30]. SPSS 19.0 (IBM Inc., Chicago, IL, USA) was employed for all data analyses except for the testing of control genotypes for Hardy–Weinberg equilibrium with the chi-square test, when Stata 14 (Stata Corp., TX, USA) was used. A 5% significance level was considered in all tests.

## Results

The study included 123 cases with GCP, 60 cases with GAgP, and 20 healthy controls. Table 1 exhibits data on age, sex, and n° cigarettes/day for each group. Table 2 displays the distribution of cases by periodontitis group and genotype. The genotype frequencies of the three studied SNPs were confirmed to be in Hardy–Weinberg equilibrium in the control group (*p* = 1.00 for IL-1A, *p* = 1.00 for IL-1B, *p* = 0.35 for IL-1RN).

**Table 1:** Patient characteristics by group.

			Total	Control (n = 20)	GAgP (n = 60)	GCP (n = 123)	p-value
Age (yrs)	≤25	N	16	1	15	0	
		%	7.88%	6.30%	93.80%	0.00%	
	26–35	N	45	12	33	0	
		%	22.17%	26.70%	73.30%	0.00%	
	36–45	N	55	2	12	41	
		%	27.09	3.60%	21.80%	74.50%	
	46–55	N	49	4	0	45	
		%	24.14%	8.20%	0.00%	91.80%	
	≥56	N	38	1	0	37	
		%	18.72%	2.60%	0.00%	97.40%	
Sex	Female	N	129	14	43	72	p = 0.183*
		%	63.55%	10.90%	33.30%	55.80%	
	Male	N	74	6	17	51	
		%	36.45%	8.10%	23.00%	68.90%	
Cigarettes/day	0	N	109		32	77	p = 0.451*
		%	59.89%		29.36%	70.64%	
	1–15	N	37		15	22	
		%	20.33%		40.54%	59.46%	
	≤16	N	36		12	24	
		%	19.78%		33.33%	66.67%	

* Chi-square test

**Table 2:** Presence of IL-1 polymorphisms and genotype distribution in the three study groups.

	Polymorphism N (%)			Polymorphism genotype N (%)	
**IL-1A (rs1800587)**	Yes	No (CC)	p-value	Heterozygous (CT)	Homozygous (TT)
**Chronic Periodontitis**	61 (58.7)	62 (62.6)	0.000*	47 (55.3)	14 (73.7)
**Aggressive Periodontitis**	40 (38.5)	20 (20.2)		35 (41.2)	5 (26.2)
**Control**	3 (2.9)	17 (17.2)		3 (3.5)	0 (0)
**IL-1B (rs1143634)**		(CC)		Heterozygous (CT)	Homozygous (TT)
**Chronic Periodontitis**	55 (56.1)	68 (64.8)	0.000*	45 (52.9)	10 (76.9)
**Aggressive Periodontitis**	41 (41.8)	19 (18.1)		38 (44.7)	3 (23.1)
**Control**	2 (2.0)	18 (17.1)		2 (2.4)	0 (0)
**IL-1RN (rs419598)**		(TT)		Heterozygous (TC)	Homozygous (CC)
**Chronic Periodontitis**	61 (64.2)	62 (57.2)	0.453*	59 (68.6)	2 (22.2)
**Aggressive Periodontitis**	24 (25.3)	36 (33.3)		20 (23.3)	4 (44.4)
**Control**	10 (10.5)	10 (9.3)		7 (8.1)	3 (33.3)

*exact p-value for distribution of responses among the three groups (chi-square test)

**Fig 1:**
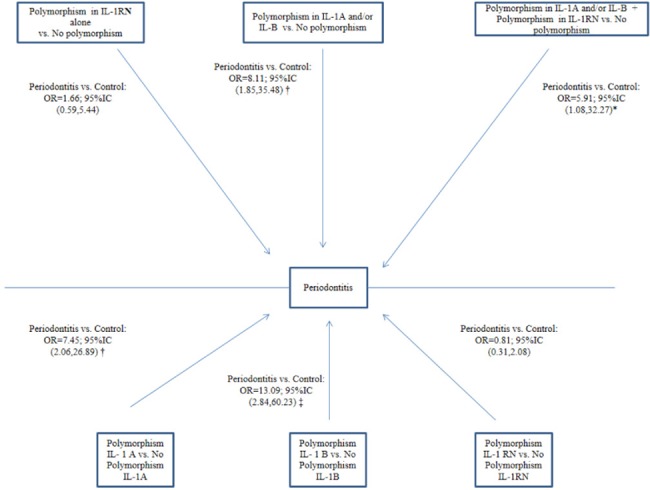
Logistic regression models of the association between polymorphisms and periodontitis. *p-value < 0.05; † p-value <0.01; ‡ p-value < 0.001. Variables from regression models adjusted for age and sex.

Sex- and age-adjusted logistic regression models were constructed, and the adjusted OR values obtained are depicted in Fig 1.

Considering GCP and GAgP results together, the likelihood of periodontitis was greater in patients with IL-1A and/or IL-1 B polymorphisms than in those with neither polymorphism (OR = 8.11, 95%CI [1.85–35.48]). No significant association was found between the presence of IL-1 RN polymorphism and the development of periodontitis (OR = 1.66; 95%CI [0.59–5.44]). A greater likelihood of periodontitis was observed for the presence *versus* absence of IL-1A polymorphism (OR = 7.45, 95%CI [2.06–26.89]), regardless of whether another polymorphism was present; similar results were observed for IL-1B, as depicted in Fig 1.

**Fig 2:**
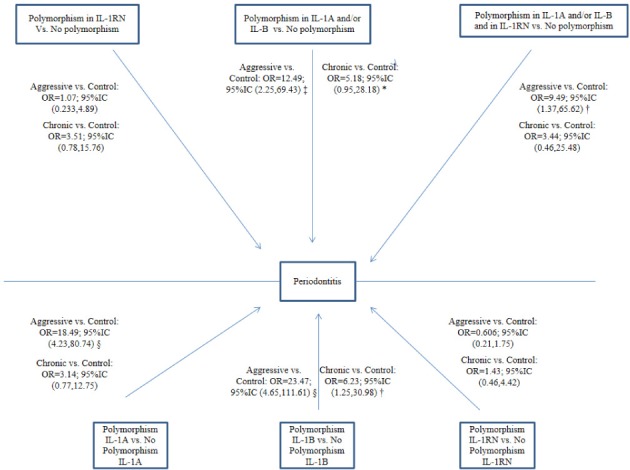
Logistic regression models of the association of polymorphisms with each type of periodontitis versus controls. *p-value < 0.1; † p-value < 0.05; ‡ p-value <0.01; § p-value < 0.001. Variables from regression models adjusted for age and gender.

Age- and sex- adjusted multinomial logistic regression models were constructed for each type of periodontitis, and Fig 2 depicts the adjusted OR, 95% CI, and p values (vs. controls) by genotype polymorphism. The likelihood of periodontitis (aggressive + chronic) was significantly higher for patients with IL-1B polymorphism (OR = 23.47; 95%CI [4.65–111.61] for GAgP and OR = 6.23; 95%CI [1.25–30.98] for GCP). The likelihood of GAgP was significantly higher for those with IL-1A polymorphism *versus* controls (OR = 18.49; 95%CI [4.23–80.74]). However, the presence of IL-1A and/or IL-1B polymorphism was not significantly associated with GCP (OR = 5.18; 95%CI [0.95–28.18]). The likelihood of GAgP was significantly higher *versus* controls in patients with polymorphisms in IL-1RN and IL-1A and/or IL-1B (OR = 9.49; 95%CI [1.37–65.62]), but it was lower in comparison to those with polymorphisms in IL-1A and/or IL-1B alone (OR = 12.49; 95%CI [2.25–69.43]) The effect of the IL-1RN polymorphism alone did not reach statistical significance (OR = 0.606; 95%CI [0.21–1.75]).

**Fig 3:**
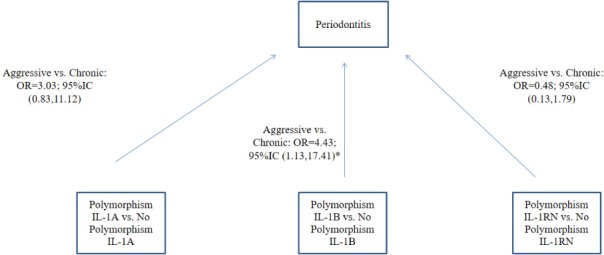
Logistic regression models of the comparison of polymorphisms between GAgP and GCP. *p-value < 0.05. Variables from regression models adjusted by age, sex and smoking. No interaction was found between smoking and genotype (p>0.1). No significant interaction of age or sex with Interleukin 1 polymorphism was found.

Logistic regression models were constructed to compare GAgP with GCP adjusted for age, sex, and smoking habit. Fig 3 depicts the OR values obtained. A significant and direct association was found between IL-1B polymorphism and GAgP (OR = 4.43; 95%CI [1.13–17.41]). No significant effect was detected for smoking habit or for the interaction between smoking and genotype.

**Fig 4:**
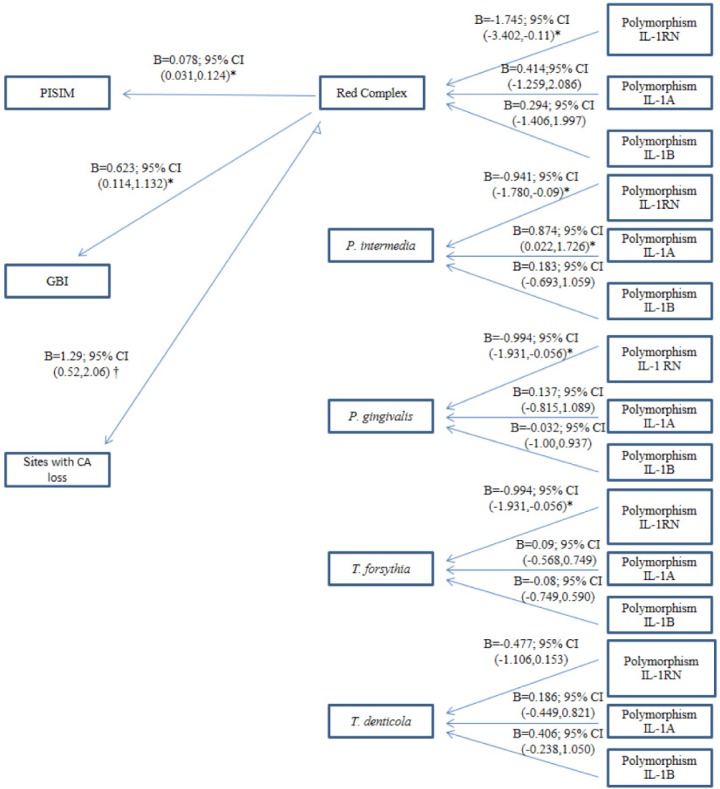
Multivariate regression analysis of the association of polymorphisms with bacterial species and clinical variables. *p-value < 0.05; † p-value < 0.001. Bacteria in log10 scale. Variables from regression models adjusted for age, sex, and smoking. No interaction between smoking and genotype was found. Non-significant relationships are not depicted.

Fig 4 shows the results of multivariate regression analysis of the association of polymorphisms with bacterial and clinical variables. In both types of periodontitis, IL-1RN polymorphism was significantly associated with a reduced load of *P*. *intermedia*, *P*. *gingivalis*, and *T*. *forsythia*, while IL-1A polymorphism was significantly associated with an increased *P*. *intermedia* load.

## Discussion

Polymorphism in the IL-1A and/or IL-1B gene was associated with a greater likelihood of the presence of periodontitis when the two types (GCP + GAgP) were considered together. The likelihood of periodontitis in patients with IL-1A and/or IL-1B polymorphism was lower when IL-1RN polymorphism was also present in comparison to IL-1A and/or IL-1B polymorphisms alone. IL-1RN polymorphism was significantly associated with a decrease in *P*. *intermedia* and in the red complex bacteria *P*. *gingivalis* and *T*. *forsythia*.

IL-1A and IL-1B gene polymorphisms have been considered a risk factor for periodontitis progression in populations of European descent due to a resulting overproduction of pro-inflammatory IL-1α and IL-1β, respectively[7, 31]. However, the contribution of IL-1RN gene and gene combinations to susceptibility to periodontitis has not been fully elucidated. In our study population, logistic regression analysis showed that polymorphism in IL-1A and/or IL-1B gene was strongly associated with generalized periodontitis (GCP or GAgP). When the polymorphism of the IL-1RN gene was also present, it exerted a protective effect against periodontitis, with a reduction in the OR from 8.11 to 5.91. The results for patients with GAgP and GCP were separately analyzed in age- and sex-adjusted multinomial logistic regression models, finding a 12.49-fold greater likelihood of GAgP in those with polymorphism in IL-1A and/or IL-1B gene than in controls and a 9.49-fold greater likelihood in those who also had a polymorphism of the IL-1RN gene.

The explanation for this protective effect of the IL-1RN gene polymorphism would be the resulting overproduction of IL-1ra. IL-1ra, an anti-inflammatory cytokine, can inhibit such IL-1α and β functions as bone resorption and connective tissue attachment loss through competitive blockade at the level of specific membrane receptors[3]. In an animal study, Nishihara et al. observed a direct dual inhibitory effect of IL-1ra, not only on the formation of osteoclast-like cells mediated by *A*. *Actinomycetemcomitans* Y4 capsular polysaccharide but also on IL-1α-induced differentiation of osteoclast progenitors into multinucleated osteoclasts [32]. Conversely, IL1-ra deficiency has been related to various infectious and inflammatory diseases, including periodontitis. Thus, a study of IL-1ra knockout mice showed a greater antigenic response to *A*. *actinomycetemcomitans* infection, with an increased production of pro-inflammatory cytokines, rapid loss of alveolar bone (assessed by micro-CT), lesser formation of mineralized matrix, and greater tissue expression of bone resorption markers[33]. Our results are compatible with the conclusion of Yucesoy et al. that an imbalance of the IL-1β/IL-1ra ratio in periodontal tissue appears to reduce susceptibility to periodontitis [34].

To our best knowledge, only three published studies have previously evaluated the mutation of the IL-1RN gene at +2018 (rs419598). Two of these, by Kornman et al. and Guzman et al., found no association between IL-1RN+2018 polymorphism and periodontitis risk in populations of European descent [15, 16]. The third study, in a Japanese population, found that individuals with this gene expression were less likely to have chronic periodontitis, with an overrepresentation in the control group, and they also defined this expression as a protective rather than risk factor for this disease[9]. Differences in IL-1 genotype distribution have been observed according to ethnicity [35]. Studies have also been performed on another mutation of this gene, VNTR allele 2 polymorphism (rs2234663), which is in linkage disequilibrium with IL-1RN+2018 (rs419598) [36]; however, findings on its relationship with periodontitis have been contradictory, as shown in the meta-analysis by Ding et al. [14].

This appears to be the first study to demonstrate a significant association of IL-1RN+2018 with reduced red complex bacteria and *P*. *intermedia* loads. IL-1RN +2018 was found to play a protective role, observing that GCP or GAgP patients with this polymorphism had a lower bacterial load of *P*. *gingivalis*, *T*. *forsythia*, and *P*. *intermedia*. By contrast, SNP‐889 (rs1800587) in IL-1A gene was associated with a significant increase in *P*. *intermedia* load. In a previous study by our group in the same population, a greater load of five periodontopathogens, including the red complex, was significantly associated with a worse clinical outcome, with higher PISIM, higher bleeding index, and more sites with CA loss ≥3 mm [17]. Hence, a reduction in the red complex implies an improvement in clinical outcomes. Our findings appear to indicate that the degree of environmental inflammation influences the growth of specific bacteria or bacterial groups. According to the ecological plaque hypothesis proposed by Marsh et al., the host environment determines the composition of resident microbiota, and changes in the oral environmental can disrupt the normal symbiotic relationship between host and resident microbes, shifting to a dysbiotic microbiota that develops in an inflammatory environment and increasing the risk of disease [37]. Immunoregulatory alterations in the host, such as SNPs, can generate favorable environmental conditions for these species because of the nutrients that come from tissue breakdown, or the host may even be less prone to dysbiosis due to an attenuated inflammatory response [38]. Our findings are compatible with this hypothesis, given that differences in the secretion of different IL-1 molecules, due to the presence of SNPs, would create a distinct inflammatory environment that would affect the microbiological composition of the subgingival plaque [39]. There is a lack of studies for comparison with the present results. Laine et al. reported a high frequency of the same IL-1A and/or IL-1B gene polymorphisms as in the present study in adults with periodontitis, although they selected the VNTR for the IL-1RN gene for investigation and did not detect *P*. *gingivalis* or *A*. *actinomycetemcomitans* by culture techniques [40].

In the present study, no significant influence on results was found for smoking habit or the interaction between smoking and genotype. Therefore, the consumption of tobacco along with the IL-1 genetic expressions studied does not appear to favor an ecosystem that increases the risk of bacterial growth and the development of periodontitis.

Study limitations include the utilization of a semiquantitative technique for pathogen counts, although this method has shown very good comparability with the widely used checkerboard DNA-DNA hybridization for the analyzed species [41]. It is also commercially available and therefore easier to apply in daily practice. Cytokine gingival crevicular fluid levels were not determined, because elevated levels have been widely correlated with IL-1 SNPs [42].

## Conclusions

In conclusion, these results indicate that IL-1RN (rs419598) gene polymorphic expression may be associated with reduced susceptibility to GAgP and GCP in populations of European descent. A reduction in the load of red complex bacteria *P*. *gingivalis*, *T*. *forsythia*, and *P*. *intermedia* would contribute to this effect.
